# Methodological progress note: Applying co‐production methods in hospital medicine research to increase impact

**DOI:** 10.1002/jhm.70464

**Published:** 2026-09-06

**Authors:** Peter J. Gill, Lisa Strifler, Francine Buchanan, Michelle Quinlan, Colin Macarthur, Sanjay Mahant

**Affiliations:** ^1^ Division of Paediatric Medicine The Hospital for Sick Children Toronto Ontario Canada; ^2^ Department of Paediatrics University of Toronto Toronto Ontario Canada; ^3^ Institute of Health Policy, Management and Evaluation University of Toronto Toronto Ontario Canada; ^4^ Child Health Evaluative Sciences, SickKids Research Institute Toronto Ontario Canada; ^5^ Office of Engagement, The Hospital for Sick Children Toronto Ontario Canada

## Abstract

Research co‐production—engaging key partners such as patients, families, clinicians, hospital administrators, and/or policymakers early and throughout the research process—enhances research impact. Through authentic, meaningful, and sustained partnerships, research co‐production helps generate evidence that is relevant and useful for patients, clinical practice, policy, and the healthcare system. This methodological progress note introduces the concept of research co‐production and provides practical strategies and real‐world examples to guide hospitalists in applying this approach to accelerate the uptake and impact of hospital medicine research.

## INTRODUCTION

Meaningful engagement that incorporates the diverse perspectives of knowledge users—patients, families, caregivers, clinicians, decision‐makers, and community members—across all stages of research ensures that evidence generated is patient‐centered, relevant, and more likely to be used in practice.^1^, ^2^ A common term used to describe this approach is research co‐production, which involves knowledge‐user partnership and collaboration across all stages of research: planning, conducting, and disseminating. When knowledge users are actively involved from the outset, their input shapes the design, interpretation, and application of findings.^3^ Other terms used to describe this approach include engagement in research, patient‐oriented research, research co‐creation and co‐design, and participatory research.^4^ In hospital medicine, potential research *partners* include patients, families, and caregivers (also referred to as lived experience advisors, patient partners), clinicians and other healthcare providers, administrative leaders, and policymakers, depending on the focus of the research.

In this methodological progress note, we introduce key concepts in research co‐production and offer practical tips, resources, and strategies for hospitalists applying co‐production methods in their research.

## CORE CONCEPTS IN RESEARCH CO‐PRODUCTION

Co‐production—engaging with knowledge users as active research partners—is perceived to improve the relevance, quality, and usefulness of research.^5^ The approach aligns with other collaborative and practice‐oriented traditions in applied health research, such as community‐based participatory research, practice‐based research networks, and learning health systems. Throughout a project, research partners are engaged in decision‐making, from identifying the problem and shaping the research question to interpreting and disseminating the results.^3^ Research co‐production emphasizes mutual respect, trust building, power sharing, inclusion, flexibility, diversity of expertise, open communication, authenticity, and equitable collaboration.^4^, ^6^, ^7^ There are several postulated benefits of co‐production including:

- Increased relevance through alignment with clinician, patient, and family priorities.
- Improved study quality through a deeper understanding of the research problem and study context.
- Increased credibility of the results.
- Enhanced recruitment and retention.
- Mutual learning and acquiring of new skills and/or knowledge.
- Relationship‐ and capacity‐building for future research.

## OPPORTUNITIES FOR RESEARCH PARTNERS TO INFLUENCE DECISION‐MAKING

A research co‐production approach is applicable to many study designs used in hospital medicine, including evidence syntheses, qualitative studies, observational studies, and randomized trials. Co‐production methods can be applied at any stage of research and involve varying levels of involvement, though early and active engagement can be helpful. Research partners do not need to participate in every phase of the research; rather, involvement should reflect their interests, preferences, and desired level of engagement. Researchers, in turn, should ensure that supportive conditions are in place to enable meaningful engagement (see Practical Considerations section). Table 1 describes opportunities where co‐production methods can engage partners and highlights concrete examples from prior *Journal of Hospital Medicine* publications.

**Table 1:** Opportunities to apply co‐production at each stage of the research process.

Research stage	Study activities	Example from hospital medicine	Reference
Research idea development and study question generation	Identifying the problem Building key relationships Setting the research priorities Shaping and refining the research question and study objectives	In the *i‐HOPE study*, patients, caregivers, clinicians and researchers identified important questions for hospitalized adult patients and created a prioritized research agenda for improving care. The *PREFER study* is co‐led by patients and clinicians, to set priorities, shape study objectives, provide input on data collection methods, and disseminate findings. Clinicians provided early feedback on the topic, grant and study protocol, and a research advisor was included as a principal investigator and co‐author. The PREFER Lived Experience Advisory Committee meets quarterly to consult on recruitment strategies and at study end to inform dissemination activities.	Harrison et al. *J Hosp Med* 2020^8^ Gill et al. *J Hosp Med* 2024^9^
Study design, including protocol development	Shaping research methods and data collection tools (e.g., systematic review inclusion criteria/screening questions, survey items, interview guides) Framing the recruitment strategy and consent forms Co‐developing and refining the intervention Supporting outcome selection and prioritization	Researchers partnered with families and hospitalists to develop the *FIQS tool*, a mobile‐responsive website for reporting safety observations. Content areas for the reports were initially based on a literature review, then narrowed via an iterative process with the hospital's Family Advisory Council and the Pediatric Hospitalist faculty.	Bardach et al. *J Hosp Med* 2022^10^
Study conduct, including data collection, analysis, and interpretation	Supporting intervention delivery and study execution Addressing challenges that arise (e.g., with recruitment) Assisting with data collection (e.g., conducting interviews) Refining data analysis plans Interpreting study findings based on lived experience	In the *Bedside Notes* study, research partners (parents, nurses, residents, hospitalists, administrators) informed the design and implementation of an intervention to enhance real‐time caregiver access to inpatient notes. Two project partners with lived experience refined the intervention educational materials and contributed to trial design and execution as members of the study steering committee.	O'Hare et al. *J Hosp Med* 2025^11^
Dissemination of research findings	Identifying audiences Crafting tailored messages from the research findings Selecting appropriate dissemination strategies Co‐designing/co‐authoring dissemination products and tools (e.g., manuscripts, infographics, video abstracts) Serving as credible messengers to share research findings Monitoring the impact of dissemination strategies	In the *Patient‐ and Family‐Centered I‐PASS program*, researchers engaged with nursing and physician leaders, family advisors, and health literacy experts as partners throughout all phases of the research. End‐of‐study knowledge mobilization activities included working with partners to disseminate the intervention and curriculum to national audiences through involvement in toolkit development, presentations, and webinars.	Khan et al. *BMJ* 2018^12^ and O'Toole et al. *J Hosp Med* 2023^13^

Abbreviations: FIQS, Family Input for Quality and Safety; i‐HOPE, Improving Hospital Outcomes through Patient Engagement; I‐PASS, Illness severity‐Patient summary‐Action items‐Situation awareness‐Synthesis by receiver; PREFER, Parenteral veRsus Enteral Fluids for infants hospitalizEd with bRonchiolitis; PIRN, Canadian Paediatric Inpatient Research Network.

## PRACTICAL CONSIDERATIONS FOR APPLYING CO‐PRODUCTION IN HOSPITAL MEDICINE RESEARCH

Figure 1 highlights practical considerations when applying a research co‐production approach to a project: begin by engaging the right research partners early, plan realistic timelines and resources to sustain meaningful engagement, review and refine approaches as the study evolves, and report on roles and activities transparently.

**Figure 1:**
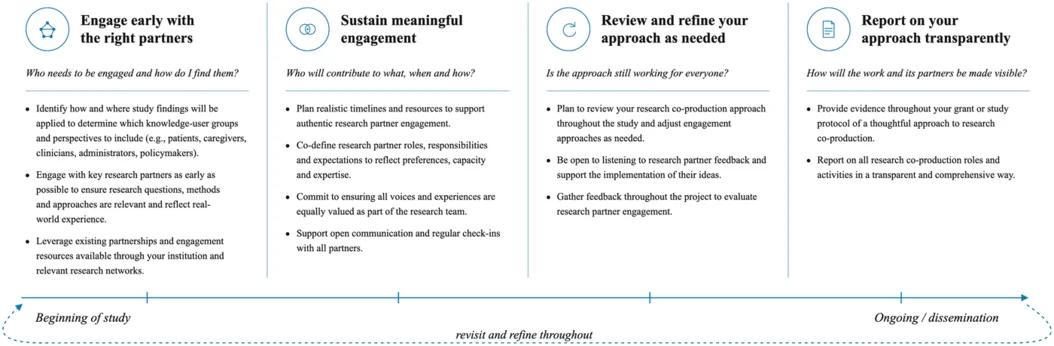
Practical considerations when applying a research co‐production approach.

### Engage early with the right research partners

Successful application of co‐production involves early engagement of partners with the appropriate lived and living experience of illness (patients/families/caregivers), practical knowledge of care delivery (clinicians and patients/families/caregivers), methodological experience (researchers), or implementation expertise (administrators/policymakers). Incorporating such perspectives from the start ensures that research questions, methods, and approaches reflect real‐world experience. It also makes the study more practical and feasible, ensures that research questions are relevant, and increases the likelihood of uptake of findings. When determining who needs to be engaged, researchers can consider how or where the research results will be applied and engage relevant individuals (e.g., clinicians and patients) as early as possible in discussions to understand context and nuance.^3^ When feasible, researchers can include partners with a broad range of lived experiences, identities, and forms of expertise. This ensures that no single individual is positioned as speaking for an entire population and that varied perspectives can shape the work. The aim is not to create a group that will agree, but to bring together people who see an issue differently so that a fuller set of needs, challenges, and priorities can emerge. For example, working with an institutional Patient and Family Advisory Council or Office of Engagement can help understand patient, family, and caregiver perspectives. Relevant research networks or support organizations, for example, the Patient‐Centered Outcomes Research Institute (PCORI) or the Strategy for Patient‐Oriented Research (SPOR)^14^, ^15^ can also facilitate connections with knowledge users.

### Support meaningful sustained engagement

Research partner engagement done poorly can erode trust and may even lead to long‐lasting harm. Common challenges and barriers to research co‐production include not investing the time and resources needed to build authentic relationships, lack of experience with engagement, and limited institutional support. Research teams may also experience logistical challenges such as scheduling meetings with research partners, communication barriers, and power imbalances. Researchers can address these challenges by planning realistic timelines and budgets for ongoing engagement from the onset, soliciting and responding to research partner feedback, involving research partners in shaping key project decisions, and appropriately remunerating research partners for their time, expertise, and expenses. Research partners should also be enabled to engage in ways that reflect their preferences and capacity. This includes co‐defining roles, responsibilities, and expectations to avoid misaligned expectations. Common roles for research partners include steering or executive committee member, advisory panel or board member, working group lead or member, engagement lead or coordinator, research team member, and consultant.^2^ Limited experience or skills in engagement in research can be supported by leveraging existing institutional or research network supports such as patient engagement offices or networks such as PCORI or SPOR.

Sustaining co‐production means showing partners how their contributions fit into a broader research agenda. When people understand the purpose of their involvement and see how their input shapes direction over time, they are more likely to stay engaged. An engagement strategy aligned with overall research goals creates continuity and shared purpose. For example, starting with a collaborative research‐prioritization process when developing a program of research can give all knowledge user groups a common foundation for future work.

Finally, maintaining partnerships over time requires a commitment to valuing different voices, sources of knowledge, and lived experiences; supporting direct and transparent communication to build trust; and being clear about how decisions are made within the project.^2^ Avoiding tokenism is central to this work: partners must have genuine opportunities to shape decisions rather than being included in name only or asked to endorse choices they had no role in influencing.

### Review and refine co‐production approaches as needed

Research teams should regularly review their research co‐production approach, solicit research partner feedback, and adapt engagement strategies as needed.^3^ For example, teams may plan end‐of‐study meetings with research partners to interpret the results, develop key messages, and co‐design dissemination products, including outputs not initially planned for in the grant. It is also beneficial to gather partner feedback throughout the project to evaluate the engagement process and knowledge mobilization goals.^2^

### Report on co‐production approaches in study protocols and grants

There are no guidelines for transparent and comprehensive reporting of research co‐production approaches. Perhaps as a result, details about co‐production roles and activities are often poorly described in research manuscripts. Transparent reporting includes when and to what extent different research partners influenced decision‐making within the study.^2^ *JHM* encourages authors to report on research co‐production in the methods section, including patient and public involvement in the research. The CONSORT (Consolidated Standards of Reporting Trials) 2025 statement has a new item recommending that authors report on how patients or the public were involved in the design, conduct, and/or reporting of the trial.

Major national research funders, such as PCORI in the United States^2^ and the CIHR SPOR in Canada,^15^ expect engagement with lived experience advisors and other research partners. These agencies also offer specific grants to support research co‐production projects and activities, including relationship‐building, planning, and dissemination. When applying for funding, it is important to describe the intention of meaningful and authentic engagement, including how research partners will provide input throughout the project. This may include descriptions of early meetings with research partners and how their perspectives have influenced the project so far, reflecting partner remuneration in a research budget, and describing plans for research partner inclusion as co‐authors on papers and other dissemination products.

### Links to additional resources

PCORI (www.pcori.org/engagement-research/engagement-resources), CIHR SPOR (www.cihr-irsc.gc.ca/e/41204.html), and Patient‐Oriented Research Curriculum in Child Health (www.porcch.ca) ^16^, ^17^ websites provide valuable resources and online training modules for researchers looking to apply co‐production, including best practices for engagement in research.

## RELEVANCE BEYOND RESEARCH

Research co‐production principles also apply to quality improvement (QI) and learning health system initiatives, as meaningful partnerships between patients and clinicians have been shown to enhance care quality, safety, and patient/family experience. For example, engaging patients, clinicians, and administrators as partners in a hospital‐based QI project or learning health system initiative can shape a better understanding of the problem, what data are gathered, how the data are used to generate new knowledge, and how that new knowledge is applied in practice to improve care and outcomes. The Patient‐Oriented Discharge Summary (PODS), co‐designed with patients and families and implemented across Ontario hospitals, is one example of how co‐production principles can be applied in QI initiatives to improve discharge communication and care transitions.^18^

## CONCLUSION

Co‐production is a recommended approach for hospitalists who wish to enhance the impact of their research project through engaging diverse perspectives early and throughout the research process. Authentic, meaningful, and sustained research partnerships through co‐production helps generate evidence that is relevant and useful for patients, clinical practice, policy, and the healthcare system, and therefore, more likely to be used in practice, programs, policy, and healthcare system delivery.

## CONFLICT OF INTEREST STATEMENT

P.J.G. has received grants from the Canadian Institutes of Health Research (CIHR), the PSI Foundation, and The Hospital for Sick Children. F.B. has received grants from CIHR. S.M. has received grants from CIHR and the PSI Foundation and is a senior deputy editor for JHM, but was not involved in the editorial review, handling, or decision‐making process for this submission.

## Data Availability

Data sharing is not applicable to this article as no datasets were generated or analysed during the current study.
